# Histopathological study using computer database of 10 000 consecutive gastric specimens: (1) benign conditions

**DOI:** 10.1093/gastro/gou093

**Published:** 2015-02-16

**Authors:** Tadashi Terada

**Affiliations:** Department of Pathology, Shizuoka City Shimizu Hospital, Shizuoka, Japan

**Keywords:** stomach, benign lesions, histopathology

## Abstract

Using a computer database, the author investigated the histopathology of 10 000 consecutive gastric specimens, taken in the last 12 years (2002–2013) at the pathology laboratory of a Japanese hospital. Re-observation of the already examined histological sections was done when the histological diagnosis and findings on the computer data base were not very obvious. The gastric specimens were identified as 8579 benign conditions and 1421 malignant lesions. The 8579 benign conditions were comprised almost normal stomach in 74 cases (0.9%), chronic gastritis in 4374 (51.0%), benign gastric peptic ulcer in 2195 (25.6%), foveolar hyperplastic polyp in 1004 (11.7%), fundic gland polyp in 421 (4.9%), adenoma in 487 (5.6%), heterotopic pancreas in 9 (0.1%), pancreatic acinar metaplasia (PAM) in 8 (0.1%), and amyloidosis in 7 (0.1%). Chronic gastritis showed lymphocytic infiltration and frequently showed erosions and intestinal metaplasia. Gastric peptic ulcer showed exudate, necrosis, active inflammation, and regenerative atypia of the epithelium. Foveolar hyperplastic polyp revealed 23 malignant changes and frequently showed dysplastic glands and intestinal metaplasia. Fundic gland polyp demonstrated cystic dilations of fundic gland ducts. Gastric adenoma showed adenomatous proliferation in the superficial mucosa and cystic dilation of the epithelium under the adenoma. Heterotopic pancreas was located in the submucosa and consisted of acinar cells, ducts, and occasionally islets. PAM was a tiny lesion in the mucosa and consisted of only pancreatic acinar cells. Amyloidosis was primary amyloidosis with positive reaction with Congo-red stain.

## Introduction

Benign lesions of the stomach include heterotopic pancreas, pancreatic acinar metaplasia (PAM), gastric adenomyoma, congenital hypertrophic pyloric stenosis, chronic gastritis, acute gastritis, collagenous gastritis, eosinophilic gastritis, granulomatous gastritis, syphilis, malakoplakia, cytomegarovirus infection, fungal infection, graft-*vs.*-host reaction, peptic ulcer, duplication, diverticula, cysts, hyperplastic polyp, adenoma, fundic gland polyp, polyposis syndrome, inflammatory fibroid polyp, Menetrier disease, and Zollinger-Ellison syndrome [1]. In the present study, 8579 benign gastric conditions were described.

## Materials and methods

The author reviewed his computer database recording 10 000 consecutive gastric specimens, accumulated in the last 12 years at the pathology laboratory of a relatively large hospital in Japan. Histological sections were examined, when appropriate. Clinical records were also reviewed in the computer system. In appropriate cases, an immunohistochemical analysis—for p53 protein (DO-7, Dako Corp, Glostrup, Denmark), cytokeratins (AE1/AE3, Dako), Ki-67 antigens (MIB1, Dako), pancreatic digestive enzymes (polyclonal, Chemicon Corp, Tomecula, USA)—was performed with the use of the Dako Envision method (Dako), as previously described [2–4].

## Results

The 10 000 gastric specimens included 8579 benign conditions and 1421 malignant lesions. In the present study, the 8579 benign conditions were investigated, made up of 8540 endoscopic biopsies and 39 gastrectomies. This benign group comprised almost normal stomach in 74 cases (0.9%), chronic gastritis in 4374 (51.0%), benign peptic gastric ulcer in 2195 (25.6%), foveolar hyperplastic polyp in 1004 (11.7%), fundic gland polyp in 421 (4.9%), adenoma in 487 (5.6%), heterotopic pancreas in 9 (0.1%), PAM in 8 (0.1%), and amyloidosis in 7 (0.1%) (Table 1).

**Table 1.** The prevalence of various lesions among 8570 benign gastric lesions

Benign lesions	No. of cases (%)
Almost normal stomach	74 (0.9)
Chronic gastritis	4374 (51.0)
Benign peptic gastric ulcer	2195 (25.6)
Foveolar hyperplastic polyp	1004 (11.7)
Fundic gland polyp	421 (4.9)
Adenoma	487 (5.6)
Heterotopic pancreas	9 (0.1)
Pancreatic acinar metaplasia	8 (0.1)
Amyloidosis	7 (0.1)

**Figure 1.**
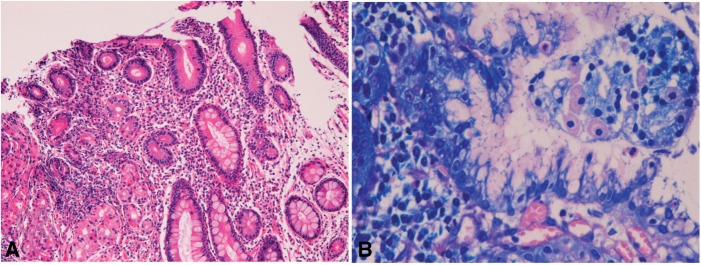
Chronic gastritis. A: Extensive lymphocyte infiltration and focal intestinal metaplasia are seen (H&E staining, ×100). (B) *Helicobactor pylori* are seen (Giemsa stain, ×400)

Chronic gastritis (*n = *4734) showed edema and lymphocytic infiltration (Figure 1A). Erosions and intestinal metaplasia were frequently observed (Figure 1A). *Helicobactor pylori* were recognized in 64% (Figure 1B). Two patients with pyloric stenosis underwent gastrectomy under the clinical diagnosis of *linitis plastica* gastric carcinoma.

**Figure 2.**
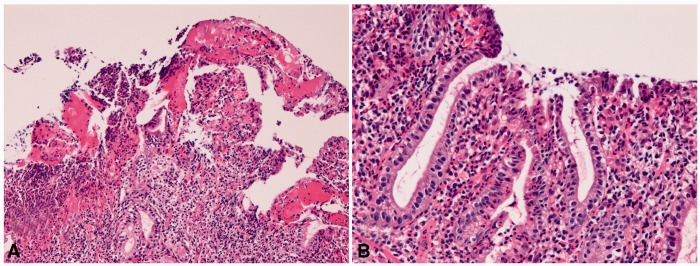
Gastric peptic ulcer. (A) Necrosis, exudate and active neurophilic and lymphocytic infiltration are recognized (H&E staining, ×100). (B) Gastric epithelium near the ulcer shows regenerative atypia (H&E staining, ×200).

Gastric peptic ulcer (*n = *2195) showed exudate, necrosis, active inflammation, and regenerative atypia of the epithelium (Figures 2A and 2B). *H. pylori* was recognized in 91% using Giemsa staining. Thirty-four patients underwent gastrectomy for recurrent ulcers.

**Figure 3.**
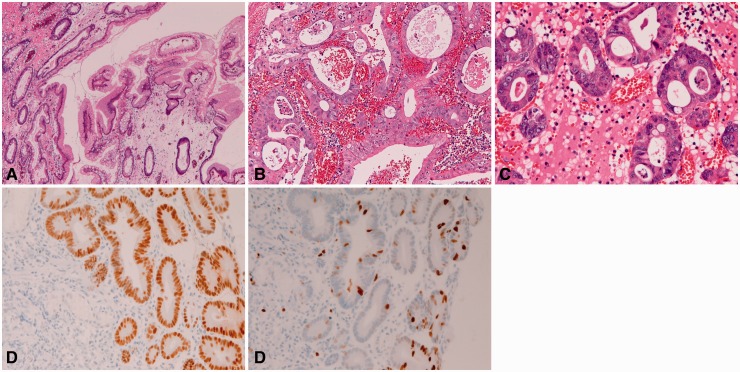
Foveolar hyperplastic polyp of the stomach. (A) Typical histological features of gastric foveolar hyperplastic polyp (H&E staining, ×40). (B) Malignant foci within gastric foveolar hyperplastic polyp (H&E staining, ×100). C: Dysplastic glands within foveolar hyperplastic polyp (H&E staining, ×100). D: p53 expression in malignant or dysplatic tubules within foveolar hyperplastic polyp (immunostaining, ×200). E: Ki-67 expression in malignant or dysplatic tubules within foveolar hyperplastic polyp (immunostaining, ×400).

Foveolar hyperplastic polyp (*n = *1004) consisted of hyperplastic foveolar epithelium and stromal edema (Figure 3A). Malignant foci of well-differentiated adenocarcinoma were recognized in 23 cases (2.3%) (Figure 3B). Dysplastic glands were identified in the vicinity of the carcinomatous foci in all these cases. Dysplastic glands without focal malignancy were identified in 104 cases (10.4%) (Figure 3C). Intestinal metaplasia was noted in 56 cases (5.6%). The malignant foci and dysplastic glands were frequently positive for p53 protein (Figure 3D) and showed a high Ki-67 labeling (Figure 3E).

**Figure 4.**
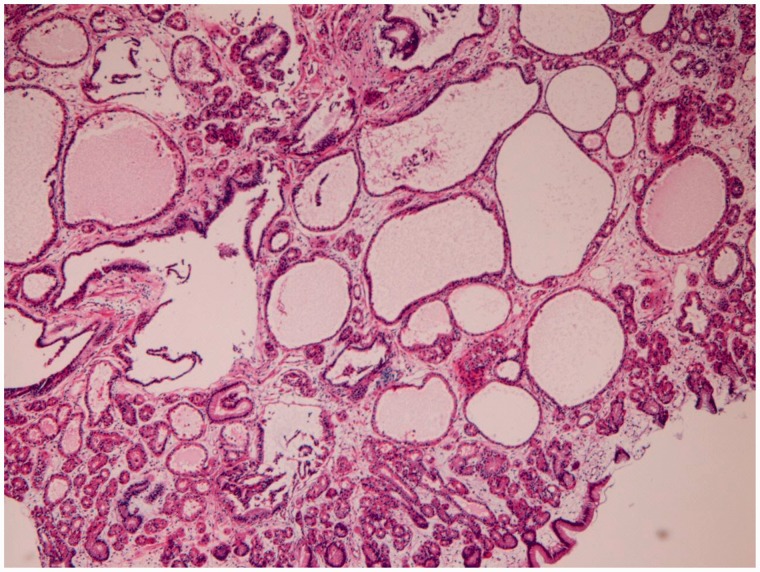
Fundic gland polyps in the stomach, showing dilations of ducts of fundic gland (H&E staining, ×40).

Fundic gland polyps (*n = *421) were small lesions, and demonstrated cystic dilation of fundic gland ducts (Figure 4). No malignant foci were recognized in the fundic gland polyps.

**Figure 5.**
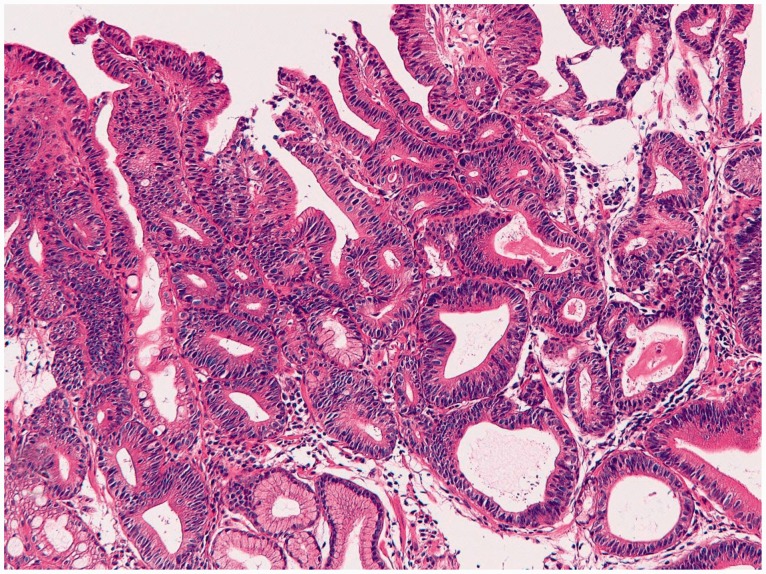
Gastric adenoma. Adenomatous proliferation is observed (H&E staining, ×100).

Gastric adenoma (*n = *487) showed adenomatous proliferation in the superficial mucosa and cystic dilation of the epithelium under the adenoma (Figure 5). No carcinomatous foci were seen, and sequential biopsies did not show malignant transformation. Twenty-nine patients underwent endoscopic mucosal resection at the patient's request.

**Figure 6.**
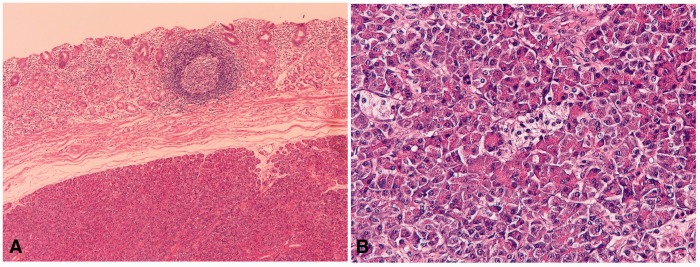
Heterotopic pancreas in the stomach. (A) Located in the submucosa (H&E staining, ×40). (B) Heterotopic pancreas is composed of acinar cell, ducts, and Langerhans islets (H&E staining, ×200).

Heterotopic pancreas (*n = *9) was located in the submucosa. Two showed cystic degeneration. Three consisted of acinar cells, ducts, and Langerhans islets (Heinrich type I) (Figure 6A), and the remaining six were composed of acinar cells and ductal elements (Heinrich type II) (Figure 6B). The pancreatic acinar cells were positive for pancreatic amylase, lipase and trypsin. Two patients underwent gastrectomy under the clinical diagnosis of gastric carcinoma.

**Figure 7.**
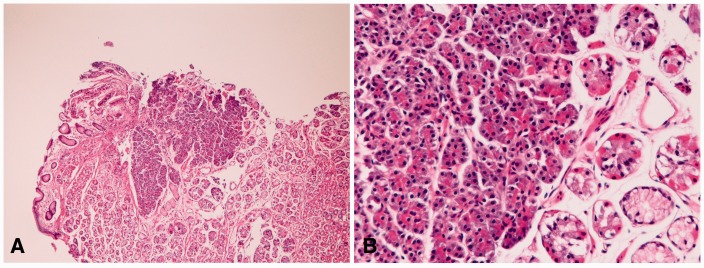
Pancreatic acinar metaplasia (PAM) in the stomach. (A) Tiny foci of pancreatic acinar cells are seen in the mucosa (H&E staining, ×40). (B) PAM is composed of only acinar cells (H&E staining, ×200).

PAM (*n = *8) was a tiny lesion in the mucosa (Figure 7A) and consisted of only pancreatic acinar cells (Figure 7B). The pancreatic acinar cells were positive for pancreatic amylase, lipase and trypsin.

**Figure 8.**
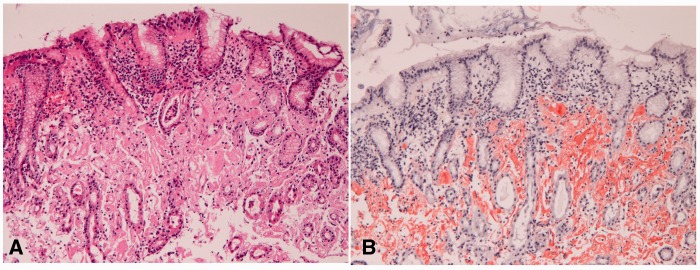
Amyloidosis in the stomach. (A) A red amorphous substance is noted in the mucosa (H&E, ×100). (B) The substance is positive with Congo-red stain (Congo-red stain, ×200).

Amyloidosis (*n = *7) was primary amyloidosis (Figure 8A) with positive reaction to Congo-red stain (Figure 8B). Six cases were associated with multiple myeloma, and the remaining one was idiopathic.

## Discussion

The most common benign gastric condition was chronic gastritis. In the author's experience as a diagnostic pathologist, most person's in Japan show lymphocytic infiltrates or chronic inflammation in the stomach. Erosions and intestinal metaplasia were frequently seen in chronic gastritis. Some patients had clinically acute gastric mucosal lesions presenting as acute gastric pain. It was interesting that two patients showed pyloric stenosis, and the clinical diagnosis of these cases was *linitis plastica* gastric carcinoma. *H. pylori* was recognized in 64% in the present series.

Gastric peptic ulcer was the second common benign condition. Pathologically, it frequently showed regenerative atypia of the epithelium. This atypia is occasionally difficult to distinguish from well-differentiated adenocarcinoma. In such cases, immunohistochemical stainings for p53 protein and Ki-67 antigen are helpful. *H. pylori* was recognized in 91% in this study.

It is very interesting that foveolar hyperplastic polyps contained malignant foci in 2.3%, and dysplastic glands in 10.0%. In the literature in English, case reports of carcinomatous foci within foveolar hyperplastic polyp have appeared sporadically [5–10]. Dysplastic changes and intestinal metaplasia within foveolar hyperplastic polyps may play an important role in the pathogenesis of the malignant transformation of such polyps [5–10]. In the present series, dysplasia–carcinoma sequence may be operative in the carcinogenesis of gastric foveolar hyperplastic polyps.

Fundic gland polyps were small lesions, characterized by cystic dilations of fundic gland ducts. This has no clinical relevance; no malignant transformation was recognized. The gastric fundic gland polyp should be differentiated endoscopically and pathologically from other gastric polypoid lesions including hyperplastic polyp, adenoma, and polypoid carcinoma.

In the present series, no malignant transformation was recognized in gastric adenoma. However, the author thinks that periodical endoscopic follow-up of patients with gastric adenoma is recommended. The most important point about adenoma is its differential diagnosis from extremely well-differentiated adenocarcinoma. This is very difficult in certain cases for pathologists.

Heterotopic pancreas is a congenital malformation [11, 12]. However, it can be misdiagnosed as gastric carcinoma and, in the present series, two patients underwent gastrectomy following clinical diagnosis of this condition. Repeated deep biopsies are necessary to obtain a correct diagnosis. Heterotopic pancreas can show cystic changes and acute hemorrhage [13–15]. Such cases shows acute abdominal pain syndrome.

PAM is a microscopic lesion in the mucosa. Unlike heterotopic pancreas, it is composed of only pancreatic acinar cells [16–19]. It has no clinical relevance, but pathologically may be misdiagnosed as adenocarcinoma. Immunohistochemical demonstration of pancreatic digestive enzymes is of great value.

Gastric amyloidosis is a gastric manifestation of systemic amyloidosis. To demonstrate the amyloid protein, Congo-red stain and immunohistochemical stainings for amyloid-related proteins are of value in the pathological diagnosis. It is necessary to detect the underlining diseases, such as multiple myeloma and chronic inflammation.

In the present study, no cases of gastric familial adenomatosis coli (FAC), gastric Crohn’s disease, or non-steroidal anti-inflammatory drugs (NSAIDs) were seen, reflecting that gastric lesions of FAC and Crohn’s disease are rare in Japan. The absence of NSAIDs may indicate that clinical information on NSAIDs was not provided to the author.

In summary, the present study reported the histopathology of various benign conditions of the stomach.
